# Scalp block for postoperative pain after craniotomy: A meta-analysis of randomized control trials

**DOI:** 10.3389/fsurg.2022.1018511

**Published:** 2022-09-26

**Authors:** Yanting Chen, Jianqiang Ni, Xiang Li, Jialei Zhou, Gang Chen

**Affiliations:** ^1^ Department of Neurology, The First Affiliated Hospital of Soochow University, Suzhou, China; ^2^ Department of Neurosurgery, The First Affiliated Hospital of Soochow University, Suzhou, China

**Keywords:** craniotomy, meta-analysis, nerve block, postoperative pain, visual analogue scale

## Abstract

**Background:**

Postoperative pain after craniotomy is an important clinical concern because it might lead to brain hyperemia and elevated intracranial pressure. Considering the side effects of opioid, several studies have been conducted to investigate the effect of local anesthetics, especially the scalp block, on postoperative pain. However, the strength of evidence supporting this practice for postoperative pain after craniotomy was unclear and the best occasion of scalp block was also not identified. Therefore, we conducted a meta-analysis to evaluate the efficacy, safety, and the best occasion of scalp block for postoperative pain after craniotomy.

**Methods:**

PubMed, Embase, and the Cochrane Library databases from database inception to October 10, 2021 were searched for all randomized controlled trials evaluating the effect of scalp block on postoperative pain after craniotomy. Data were assessed by StataMP 16 software.

**Results:**

A total of 12 studies were included. A random-effect model was used to analyze all data. Patients under scalp block earned fewer scores than the non-scalp block group in visual analogue scale at the very early period (MD = −1.97, 95% CI = −3.07 to −0.88), early period (MD = −1.84, 95% CI = −2.95 to −0.73) and intermediate period (MD = −1.16, 95% CI = −1.84 to −0.49). Scalp block could also significantly prolong the time of the first request of rescue analgesia and reduce the use of additional analgesics without a significant difference in the incidence of complications. Subgroup analysis showed there was no significant difference in analgesia effect between pre-incision scalp block and post-incision scalp block in all periods.

**Conclusion:**

Scalp block could lead to lower pain intensity scores, more time of the first request of rescue analgesia, and fewer analgesic drugs applied in the first 12 h after craniotomy. There was no significant difference between pre-incision and post-incision scalp block in the occurrence and severity of postoperative pain.

## Introduction

Craniotomy is an effective treatment of cerebral diseases and injuries, and postoperative pain is an important clinical concern. 86% of patients probably had the pain of somatic origin, with the involvement of soft tissues and pericranial muscles (1). Elevated oxygen consumption and catecholamine release caused by postoperative pain lead to brain hyperemia and elevated intracranial pressure, which may predispose them to intracranial hematoma (2–4). Effective pain management and prevention are important to avoid these systemic changes and improve rehabilitation and long-term outcomes (5, 6). Besides, management of early postoperative pain can prevent the development of central sensitization and chronic pain states caused by surgical tissue damage (7, 8). However, pain after a craniotomy is often treated insufficiently, because of the fear that the opioid-induced sedation and miosis will mask neurological pathology. Therefore, several studies have been conducted to investigate the effect of local anesthetics, especially the scalp block, on postoperative pain (9–12).

Scalp block was a common technique in craniotomy and was widely used to reduce the hemodynamic response and incisional pain during craniotomy procedure (13–15). Analgesia could be achieved by blockade of the following nerves: greater and lesser occipital nerves, the supraorbital and supratrochlear nerves, the zygomaticotemporal nerve, the auriculotemporal nerve, and the greater auricular nerve (16–18). However, the strength of evidence supporting this practice was unclear and the best occasion of scalp block was also not identified (19–21). Therefore, we conducted a meta-analysis to evaluate the efficacy, safety, and the best occasion of scalp block for postoperative pain after craniotomy.

## Methods

A meta-analysis was conducted according to the Preferred Reporting Items for Systematic Reviews and Meta-Analyses (PRISMA) guidelines. As our study was based on published literature data, ethical approval or patient consent was not required. Besides, only studies reporting ethical approval or patient consent were included in our meta-analysis.

### Search strategy

In this meta-analysis, 2 investigators performed a systematic literature search using keywords “scalp block” and “craniotomy” in PubMed, Embase, and the Cochrane Library databases from database inception to October 10, 2021, to identify randomized clinical trials that reported scalp block vs. non-scalp block in patients scheduled for craniotomy. To avoid omissions, the reference lists of all relevant articles were manually searched.

### Inclusion and exclusion criteria

Inclusion criteria were defined as follows: (1) Study type: only randomized controlled trials; (2) Population: patients aged >18 years; (3) Intervention: scalp block; (d) Studies that reported at least one of the following outcome measures: pain intensity measured by the visual analogue score, time of the first request of rescue analgesia, additional analgesia requirement in the first 24 h, and adverse events in the first 24 h.

Exclusion criteria were designed as follows: (1) Types of articles: reviews, case reports and retrospective studies; (2) Any study using methods other than placebo and systemic analgesia in the control group; (3) Any study had scalp block in all arms.

### Data extraction

Two investigators extracted the following data from the included studies: (1) Number of patients; (2) occasion of scalp block; (3) Postoperative pain treatment modality (occasion and the dosage); (4) Pain intensity for all time points; (5) Time of the first request of rescue analgesia; (6) Additional analgesia requirement in the first 24 h; (7) adverse events in the first 24 h. Data was recorded on a dedicated data extraction form.

### Outcome measures

The primary outcome was pain intensity measured by the visual analogue score within 48 h postoperative period. The interval of 0 to 100 pain intensity of VAS was rescaled to a standard interval of 0 to 10. In this study, pain level measurements were categorized into five time periods: (1) very early: < 2 h; (2) early: ≥2 h but <6 h; (3) intermediate: ≥6 h but <12 h; (4) late: ≥12 h but <24 h; (5) very late: ≥24 h but ≤48 h. When there were multiple intervention groups, all relevant experimental intervention groups or relevant control intervention groups were combined into one group. The average of their mean and standard deviation had been calculated at the same time.

### Subgroup analysis

Subgroup analysis was conducted to evaluate efficacy in two subgroups. According to the occasion of scalp block, subgroups were classified as pre-incision scalp block and post-incision scalp block.

### Risk of bias

Reviews Manager 5.4 software was used to evaluate the risk of bias in each study. Two investigators used the uniform criteria of the Cochrane collaboration for assessing the risk of bias, including selection bias, performance bias, detection bias, reporting bias, performance bias, detection bias, attrition bias, reporting bias, and other potential biases. The result of each aspect was divided into low risk, high risk, or unknown risk.

### Statistical analysis and data synthesis

Data were assessed by StataMP 16 software. We defaulted to analyzing continuous or dichotomous outcomes as mean difference (MD) or the odds ratio (OR) using a 95% confidence interval (CI) separately. Standard mean difference (SMD) was used for additional analgesia requirements in first 24 h because the specific drug and route of administration varied among trials. Heterogeneity was classified as moderate (*I*^2 ^= 25%–50%), substantial (*I*^2 ^= 50%–75%) or considerable (*I*^2 ^≥ 75%) (22). Owing to the degree of heterogeneity found, a random-effect model was used (23). Subgroup analysis was performed to investigate the stability of the consolidated results. *P*-value <0.05 was considered as significant. To determine the source of heterogeneity and assess the influence of every single study on the final results, we excluded one study in sequence through sensitivity analysis.

## Results

### Study selection

**Figure 1:**
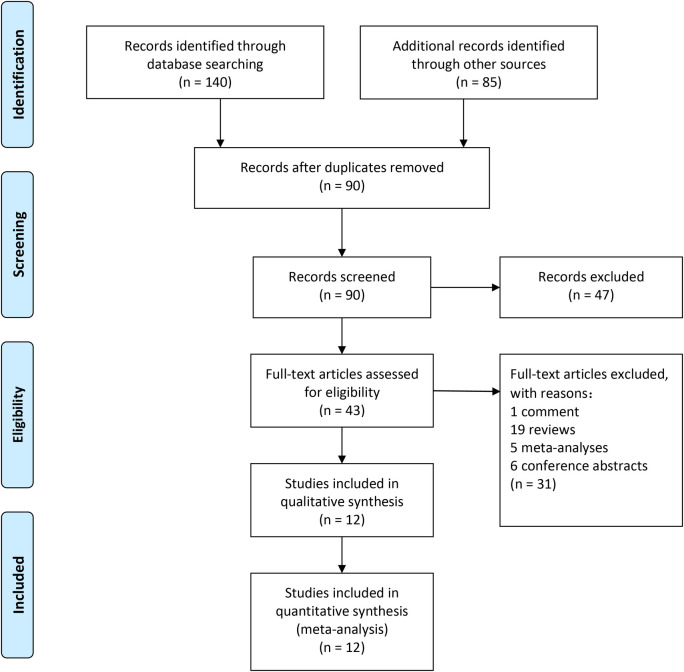
Study flow chart (as per PRISMA guideline).

**Figure 2:**
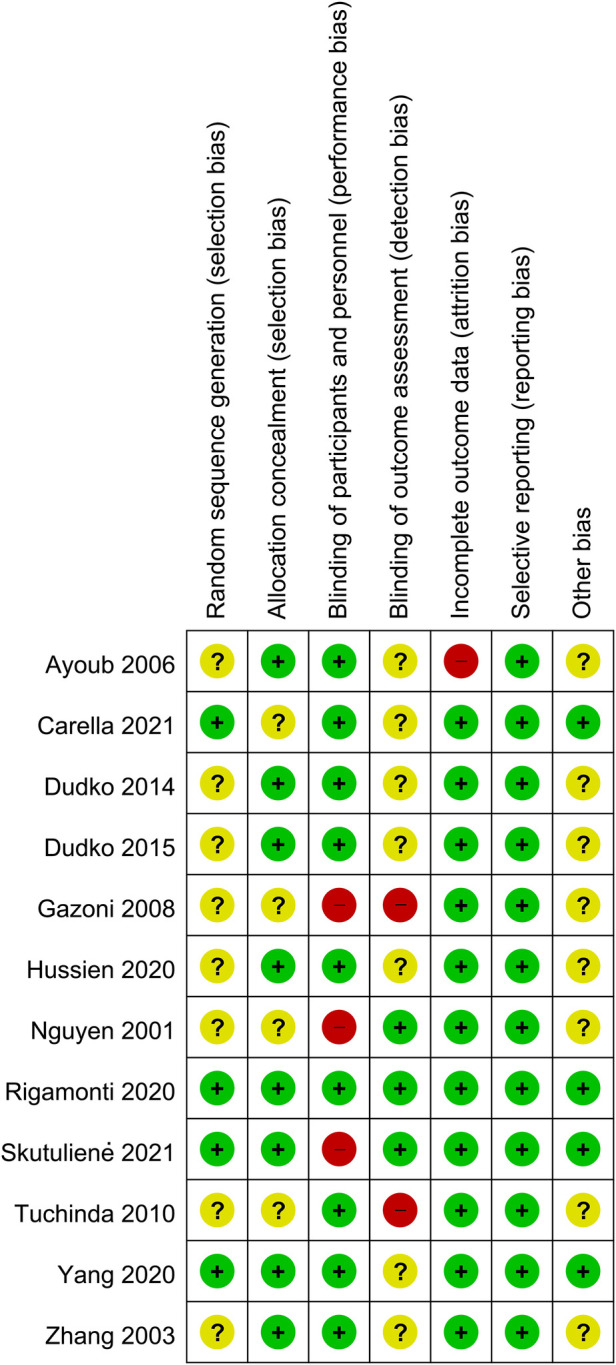
Risk of bias assessment summary.

A total of 225 articles from PubMed, Embase, and Cochrane Library databases were identified. 182 records were removed because of duplication or irrelevance to the study. 31 other records were excluded because they were comments (1 record), reviews (19 records), meta-analyses (5 records), and conference abstracts (6 records). The entire process of study search, selection, and inclusion are shown in Figure 1. The overall risk of bias in studies included is presented in Figure 2.

### Study characteristics

Details of the included studies are shown in Table 1. Full information on included studies could be accessed in the supplement. 8 trials (24–31) were analyzed for pain intensity outcome, 9 trials (4, 24–29, 32, 33) for additional analgesia outcome, and 5 trials (4, 25, 29, 33, 34) for adverse events outcome. Four trials had a three-arm design and two trials had a four-arm design. Data from all doses of scalp block in the same trial were combined as one experimental intervention group (28, 34). Several drugs were used for rescue analgesia, including ketorolac, paracetamol, pethidine, morphine, dezocine, hydromorphone, fentanyl, pethidine, morphine, and codeine phosphate.

**Table 1:** Characteristics of included trials.

Study	Year	Participants	Study type	SB	NSB	Occasion	Scale	Pain score assessments (h)	Rescue analgesia
Skutulienė et al.	2021	141	RCT	0.25% bupivacaine, 1% lidocaine and 1:200,000 epinephrine	Paracetamol 1 g and ketoprofen 2 mg/kg intravenous	Post-incision	VAS (0–100)	1, 3, 6, 24	Ketorolac, paracetamol and pethidine
Carella et al.	2021	60	RCT	0.33% levobupivacaine	Placebo	Pre-incision	VAS (0–10)	1, 3, 6, 24, 48	Morphine
Yang et al.	2020	88	RCT	0.2%, 0.33% or 0.5% ropivacaine	Placebo	Pre-incision	VAS (0–10)	2, 4, 6, 24	Dezocine
Rigamonti et al.	2019	89	RCT	0.5% bupivacaine with 1:200,000 epinephrine	Placebo	Post-incision	VAS (0–100)	1, 2, 4, 8, 12, 18, 24, 48	Hydromorphone
Hussien et al.	2020	30	RCT	0.5% bupivacaine, 2% lidocaine and 1:200,000 epinephrine	Fentanyl intravenous	Pre-incision	VAS (0–10)	0.5, 1, 2, 4, 8, 16, 24	Fentanyl or ketorolac
Dudko et al.	2015	120	RCT	0.25% bupivacaine, 1% lidocaine and 1:20,0000 adrenaline	Paracetamol 1 g and ketoprofen 2 mg/kg intravenous	Post-incision	VAS (0–100)	1, 3, 6, 24	Ketorolac, paracetamol and pethidine
Dudko et al.	2014	75	RCT	0.25% bupivacaine, 1% lidocaine and 1:200.000 adrenaline	Paracetamol 1 g and ketoprofen 2 mg/kg intravenous	Post-incision	VAS (0–100)	1, 3, 6, 24	Ketorolac, paracetamol and pethidine
Tuchinda et al.	2010	60	RCT	0.5% or 0.25% bupivacaine with 1:200,000 adrenaline	Placebo	Pre-incision	VAS (0–10)	1, 1.5, 2, 6, 12, 24	Morphine
Gazoni et al.	2008	30	RCT	0.5% ropivacaine	Standard treatment	Pre-incision	VAS (0–10)	1, 2, 4	Morphine
Ayoub et al.	2006	50	RCT	2% lidocaine and 0.5% bupivacaine	Placebo	Post-incision	NRS (0–10)	1, 2, 4, 8, 12, 16, 24	Codeine phosphate
Zhang et al.	2003	60	RCT	0.75% ropivacaine	Placebo	Post-incision	VAS (0–10)	4, 8, 12, 16, 20, 24, 48	N/A
Nguyen et al.	2001	30	RCT	0.75% ropivacaine	Placebo	Post-incision	VAS (0–10)	4, 8, 12, 16, 20, 24, 48	N/A

Abbreviation: SB, scalp block; NSB, non-scalp block; RCT, randomized controlled trial; VAS, visual analogue scale; NRS, numerical rating scale; N/A, not available in report.

### Outcomes

The summary of the meta-analysis results was in Table 2.

**Table 2:** Summary of meta-analysis results.

Outcome or Subgroup	Studies	Participants	Statistical Method	Effect Estimate
1. Very early VAS	6	446	Mean Difference (IV, Random, 95% CI)	−1.97 [−3.07, −0.88]
1.1 Pre-incision scalp block	4	331	Mean Difference (IV, Random, 95% CI)	−2.03 [−3.53, −0.53]
1.2 Post-incision scalp block	2	135	Mean Difference (IV, Random, 95% CI)	−1.87 [−3.92, 0.18]
2. Early VAS	7	456	Mean Difference (IV, Random, 95% CI)	−1.84 [−2.95, −0.73]
2.1 Pre-incision scalp block	4	229	Mean Difference (IV, Random, 95% CI)	−1.87 [−3.51, −0.23]
2.2 Post-incision scalp block	3	227	Mean Difference (IV, Random, 95% CI)	−1.67 [−3.05, −0.29]
3. Intermediate VAS	7	331	Mean Difference (IV, Random, 95% CI)	−1.16 [−1.84, −0.49]
3.1 Pre-incision scalp block	3	139	Mean Difference (IV, Random, 95% CI)	−0.88 [−2.23, 0.46]
3.2 Post-incision scalp block	4	192	Mean Difference (IV, Random, 95% CI)	−1.31 [−2.03, −0.59]
4. Late VAS	5	430	Mean Difference (IV, Random, 95% CI)	−0.98 [−2.13, 0.17]
4.1 Pre-incision scalp block	2	89	Mean Difference (IV, Random, 95% CI)	−0.45 [−1.35, 0.46]
4.2 Post-incision scalp block	3	341	Mean Difference (IV, Random, 95% CI)	−1.39 [−3.32, 0.53]
5. Very late VAS	7	523	Mean Difference (IV, Random, 95% CI)	−1.09 [−2.22, 0.04]
5.1 Pre-incision scalp block	3	189	Mean Difference (IV, Random, 95% CI)	−0.70 [−2.06, 0.67]
5.2 Post-incision scalp block	4	334	Mean Difference (IV, Random, 95% CI)	−1.45 [−3.47, 0.57]
6. Time of the first request of rescue analgesia	5	313	Mean Difference (IV, Random, 95% CI)	164.65 [65.28, 264.01]
6.1 Pre-incision scalp block	2	89	Mean Difference (IV, Random, 95% CI)	46.69 [−75.61, 168.98]
6.2 Post-incision scalp block	3	224	Mean Difference (IV, Random, 95% CI)	282.48 [67.17, 497.79]
7. Additional analgesia requirement in first 24 h	7	354	Std. Mean Difference (IV, Random, 95% CI)	−0.88 [−1.62, −0.13]
7.1 Pre-incision scalp block	4	169	Std. Mean Difference (IV, Random, 95% CI)	−1.58 [−2.92, −0.24]
7.2 Post-incision scalp block	3	185	Std. Mean Difference (IV, Random, 95% CI)	−0.13 [−0.71, 0.45]
8. Nausea and vomiting in first 24 h	5	344	Odds Ratio (M-H, Random, 95% CI)	0.61 [0.23, 1.67]
8.1 Pre-incision scalp block	2	115	Odds Ratio (M-H, Random, 95% CI)	0.47 [0.04, 5.65]
8.2 Post-incision scalp block	3	229	Odds Ratio (M-H, Random, 95% CI)	0.75 [0.25, 2.22]

Abbreviation: VAS, visual analogue scale; CI, confidence interval. Numbers in bold indicate a significant treatment effect (*P* < 0.05).

#### Visual analogue scale

**Figure 3:**
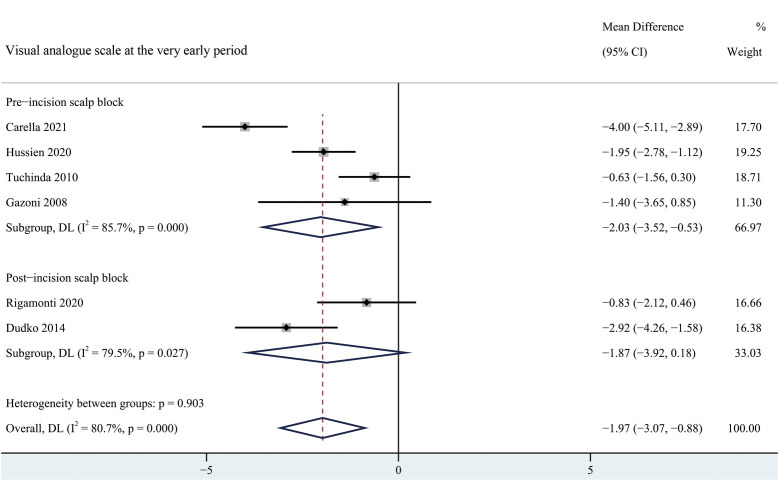
Forest plot summarizing meta-analysis of studies reporting very early pain score. Notes: The black solid rhombuses indicate the estimated mean difference for each randomized controlled trial and the extending lines indicate the estimated 95% CI of mean difference for each randomized controlled trial; The black hollow rhombuses indicate the estimated mean difference (95% CI) for patients in each subgroup or all patients included; Weights are from a random-effects analysis. Abbreviation: SB, scalp block; NSB, non-scalp block; CI, confidence interval.

There was lower pain intensity in patients receiving scalp block at the very early period (MD = −1.97, 95% CI = −3.07 to −0.88), early period (MD = −1.84, 95% CI = −2.95 to −0.73) and intermediate period (MD = −1.16, 95% CI = −1.84 to −0.49) than the non-scalp block group (Figure 3, Supplementary Figures S1–S2). No significant reduction in reported pain scores associated with scalp bock was found at late or very late period (Supplementary Figures S3–S4).

#### Rescue analgesia

Six studies reported the time of the first request of rescue analgesia and there was an overall increment associated with scalp bock (MD = 164.65, 95% CI = 65.28 to 264.01). Scalp block led to a notably decrease on additional analgesia requirement in first 24 h (SMD = −0.88, 95% CI = −1.62 to −0.13).

#### Adverse events

**Figure 4:**
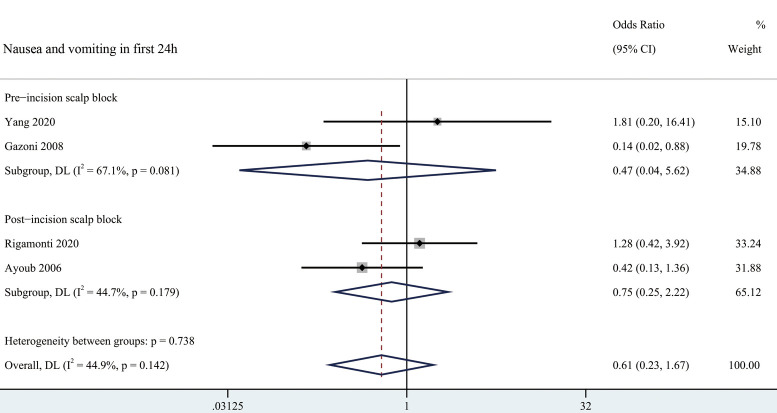
Forest plot summarizing meta-analysis of studies reporting nausea and vomiting in first 24 h. Notes: The black solid rhombuses indicate the estimated odds ratio for each randomized controlled trial and the extending lines indicate the estimated 95% CI of odds ratio for each randomized controlled trial; The black hollow rhombuses indicate the estimated odds ratio (95% CI) for patients in each subgroup or all patients included; Weights are from a random-effects analysis. Abbreviation: SB, Scalp Block; NSB, non-Scalp Block; CI, confidence interval.

Nausea and vomiting odds ratio in the first 24 h related to scalp block was 0.61 (95% CI = 0.23 to 1.67) as shown in Figure 4. None of the included trials reported any significant difference in the incidence of other complications such as local hematoma, infection, or nerve injury (24, 26–28, 30–32).

#### Subgroup analysis

According to the occasion of scalp block, patients were divided into pre-incision scalp block group and post-incision scalp block group. Pre-incision scalp block was significantly effective at the very early and early period, whereas pre-incision scalp block showed a significant reduction in pain scores at early and intermediate period after surgery (Figure 3, Supplementary Figures S1–S4). There was no significant difference in the time of the first request of rescue analgesia between pre-incision scalp block and post-incision scalp block (Supplementary Figure S5). Besides, a significant reduction in the usage of analgesia requirement was found in the post-incision scalp block group (Supplementary Figure S6).

### Study bias assessment

Egger's publication bias plot was used to assess the potential risk of publication bias Supplementary Figure S7) and no significant funnel plot asymmetry was observed. Results of “leave-one-out” sensitivity analysis are provided in Supplementary Figures S8–S15.

## Discussion

This meta-analysis of 12 randomized controlled trials reviewed the available evidence to evaluate the efficacy and safety of scalp block for postoperative analgesia after craniotomy. Combining all studies, we found scalp block was effective in reducing short-term pain without increasing the risk of associated complications, no matter whether it was used before or after incision.

Postoperative pain after craniotomy is mainly localized in the incision and surrounding soft tissues and is less likely to be a widespread headache. The pathophysiological mechanisms of postoperative pain involve different pathways of injury perception. The effects of different analgesic techniques acted on these injury perception pathways. To block the scalp innervation and to anesthetize both the superficial layers of the scalp, local anesthetic infiltration has been accepted by many neurosurgeons, but the effect is short-lived. A study conducted by Akcil et al. (35) found anesthetic infiltration could provide effective anesthesia only in the first 10 min postoperatively, compared with systemic anesthesia. Our study showed a significant mean reduction in pain score at up to 12 h after craniotomy and the analgesic effect decreased over time. In general, the effect of local anesthetic lasts no more than 6 h. The effect of scalp block seemed to persist longer than expected, considering the duration of craniotomy. This finding might be explained by the preemptive analgesic effect. When scalp block is administered, it blocks the C fiber as well as prevents part of the inflammatory cascade, which could reduce or even eliminate the pain hypersensitivity.

Rescue analgesia is usually applied when the pain score is above a certain value, or the pain is unbearable. The longer time of first request of rescue analgesia (MD = 164.65, 95% CI = 65.28 to 264.01) and the less usage of additional analgesics (SMD = −0.88, 95% CI = −1.62 to −0.13) were found in our study. The fewer additional analgesics used postoperatively, the associated side effects like gastrointestinal bleeding caused by NSAIDs or ventilation depression caused by opioids are less likely to occur.

Concerns about postoperative pain management revolved around the side effects of sedation, miosis, nausea, and vomiting, which could probably mask some crucial clinical symptoms. Expect for a trial of Gazoni et al. (29), none of the other trials included reported any significant difference in the incidence of complications. Analysis of 5 randomized controlled trials including a total of 190 participants demonstrated that no significant variation could be found in the complications, with substantial heterogeneity. We believed that scalp block was equally safe compared to traditional analgesic methods.

We conducted a subgroup analysis to figure out the advantages and disadvantages of the different occasion of scalp block and found that there was no significant difference in analgesia effect between pre-incision scalp block and post-incision scalp block. However, we found different periods of postoperative analgesia: pre-incision scalp block was effective for analgesia at very early and early periods, but post-incision scalp block was better at the early and intermediate periods. Considering the same duration of effect of scalp block, it's important to allow effective time to cover the most critical periods. When only considering the postoperative analgesic effect, performing scalp block postoperatively was recommended. However, pre-incision scalp block might also have the advantage of blunting the hemodynamic response to noxious stimuli such as cranial stapling, skin dissection, and flap stripping, which is of great importance when patients are scheduled for awake craniotomies. In general, the occasion of scalp block should be determined upon which purpose the neurosurgeons want to achieve. We believed that pre-incision scalp block should be applied when better intraoperative analgesia was needed, otherwise post-incisional scalp block was more reliable.

Overall, Scalp block is a safe and reliable technique that can effectively reduce patients' pain in the first 12 h after craniotomy, and the results of our study supported its use in postoperative analgesia after craniotomy.

Several limitations were objectively included in this study. First, only 12 randomized controlled studies were included in the study and no more data were available to support our conclusions. Secondly, there were also methodological differences among the included trials, such as different types and doses of local anesthetics. Thirdly, we did not focus on the relevance between the duration of craniotomy and the effect of scalp block on postoperative pain after craniotomy. We hope more trials and studies related to scalp block could be conducted in the future to support our conclusion.

## Data Availability

The raw data supporting the conclusions of this article will be made available by the authors, without undue reservation.
