# Supplementary material for: Scalp block for postoperative pain after craniotomy: A meta-analysis of randomized control trials
Source: Front Surg. 2022 Sep 26;9:1018511. doi: 10.3389/fsurg.2022.1018511 (PMC9550001; doi:10.3389/fsurg.2022.1018511)
Supplement: Supplementary file 1 [file DataSheet1.pdf]

Supplement

Supplemental Figure 1. Forest plot summarizing meta-analysis of studies reporting early pain score.. 2

Supplemental Figure 2. Forest plot summarizing meta-analysis of studies reporting intermediate pain score................................ 3

Supplemental Figure 3. Forest plot summarizing meta-analysis of studies reporting late pain score.. 4

Supplemental Figure 4. Forest plot summarizing meta-analysis of studies reporting very late pain score...................................... 5

Supplemental Figure 5. Forest plot summarizing meta-analysis of studies reporting time of the first request of rescue analgesia.. 6

Supplemental Figure 6. Forest plot summarizing meta-analysis of studies reporting additional analgesia requirement in first 24h.7

Supplemental Figure 7. Summary of the Egger's publication bias plot. 8

Supplemental Figure 8. "Leave-one-out" sensitivity analysis of studies reporting very early pain score. 9

Supplemental Figure 9. "Leave-one-out" sensitivity analysis of studies reporting early pain score. 10

Supplemental Figure 10. "Leave-one-out" sensitivity analysis of studies reporting intermediate pain score................................ 11

Supplemental Figure 11. "Leave-one-out" sensitivity analysis of studies reporting late pain score.. 12

Supplemental Figure 12. "Leave-one-out" sensitivity analysis of studies reporting very late pain score...................................... 13

Supplemental Figure 13. "Leave-one-out" sensitivity analysis of studies reporting time of the first request of rescue analgesia.. 14

Supplemental Figure 14. "Leave-one-out" sensitivity analysis of studies reporting additional analgesia requirement in first 24h.15

Supplemental Figure 15. "Leave-one-out" sensitivity analysis of studies reporting nausea and vomiting in first 24h.................. 16

Characteristics of included studies (order by year of publication) 17

Supplemental Figure 1. Forest plot summarizing meta-analysis of studies reporting early pain score.

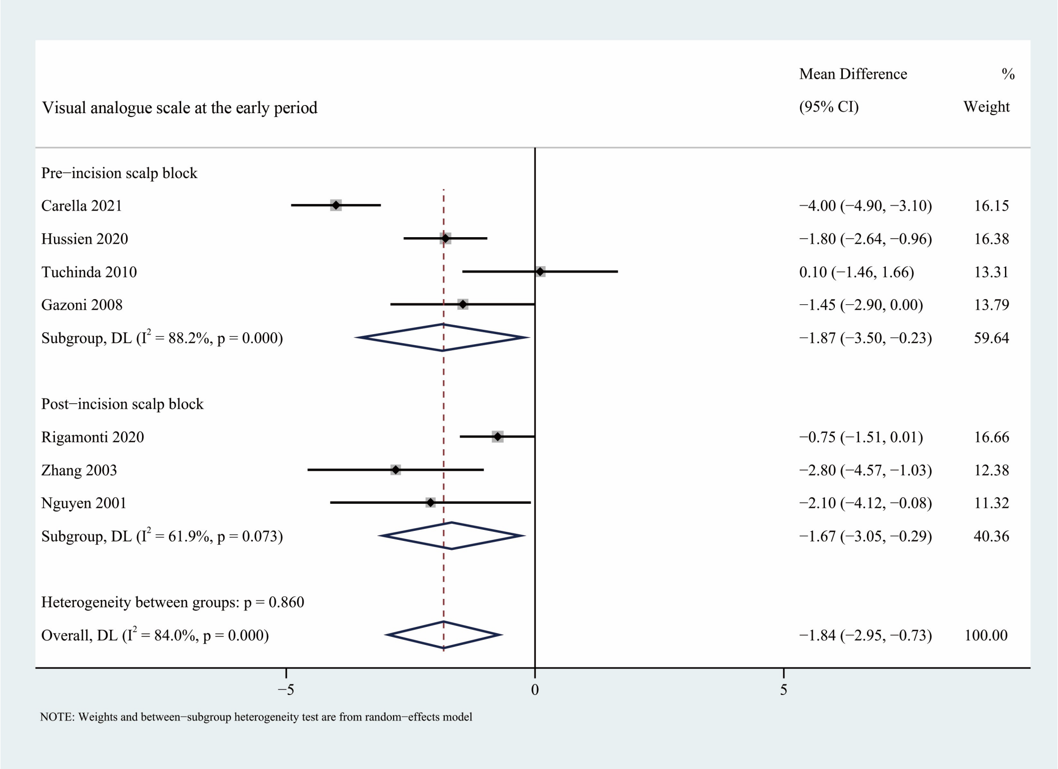

Supplemental Figure 2. Forest plot summarizing meta-analysis of studies reporting intermediate pain score.

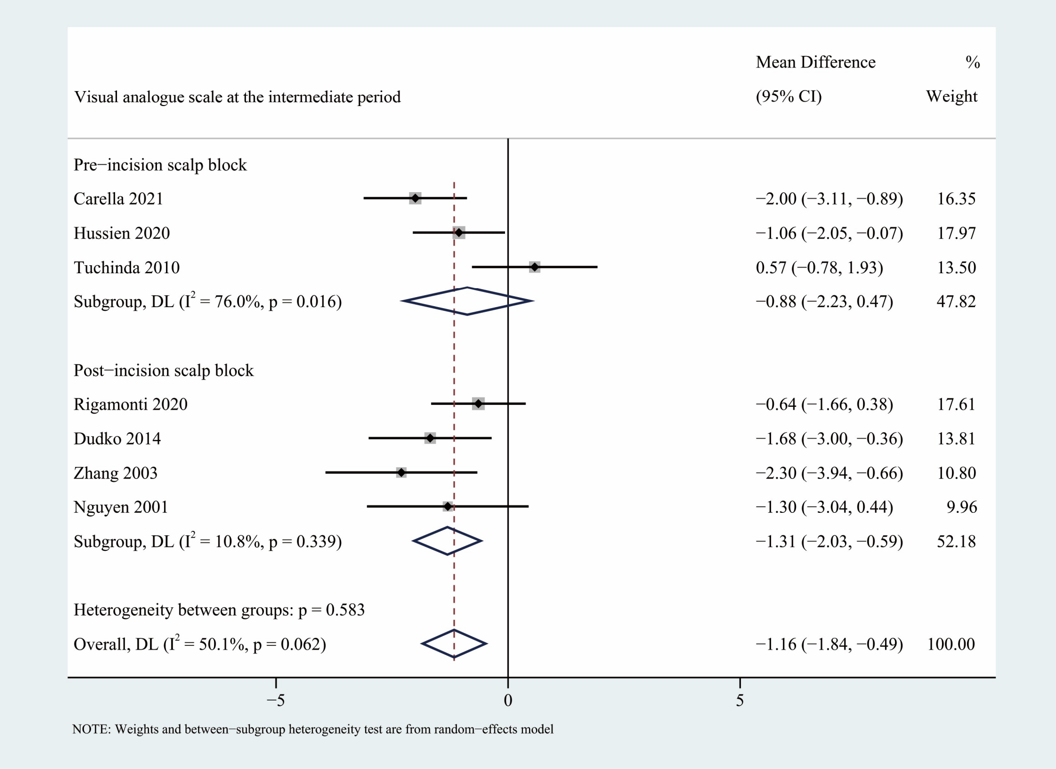

Supplemental Figure 3. Forest plot summarizing meta-analysis of studies reporting late pain score.

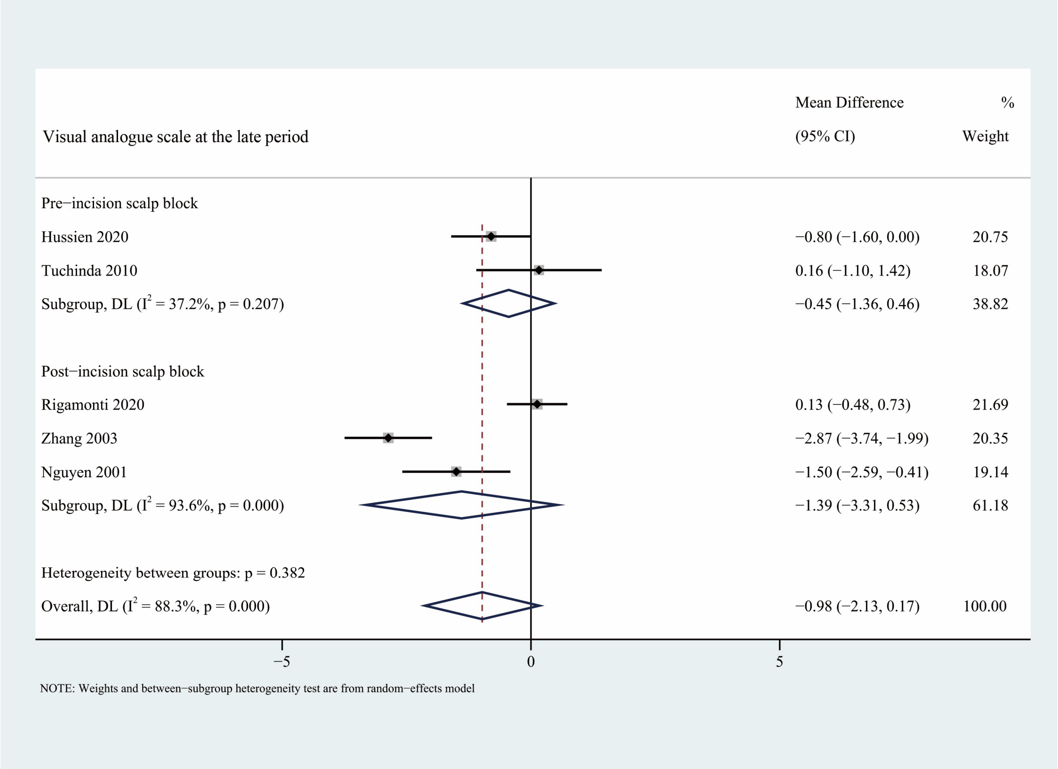

Supplemental Figure 4. Forest plot summarizing meta-analysis of studies reporting very late pain score.

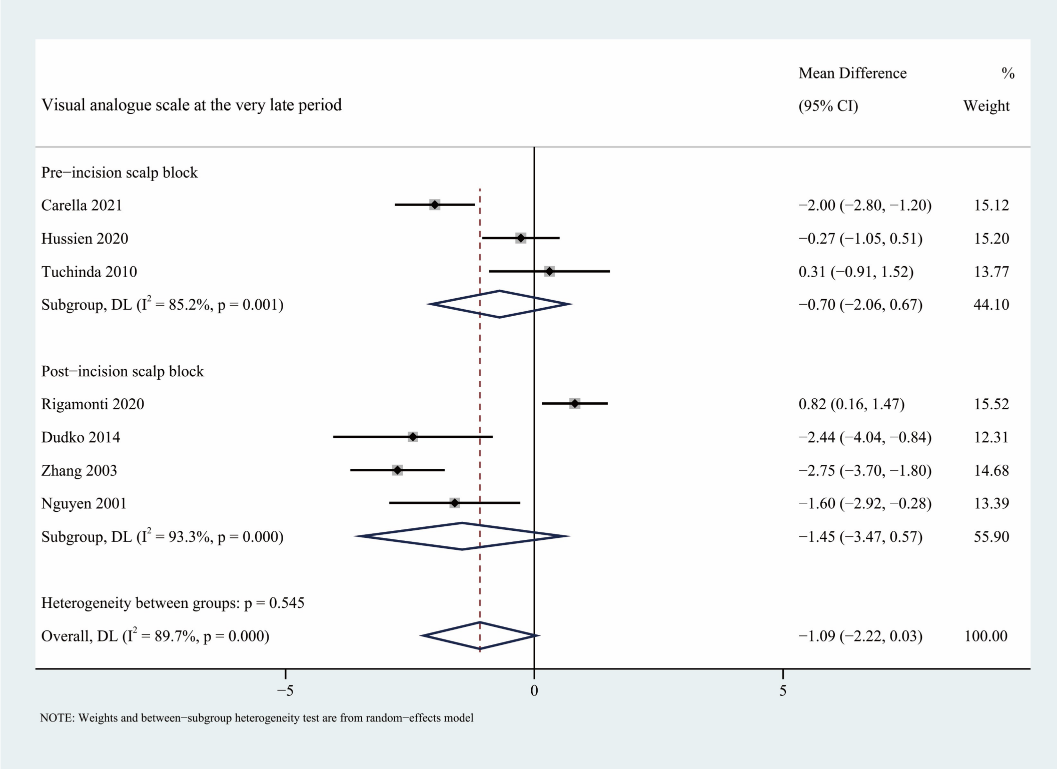

Supplemental Figure 5. Forest plot summarizing meta-analysis of studies reporting time of the first request of rescue analgesia.

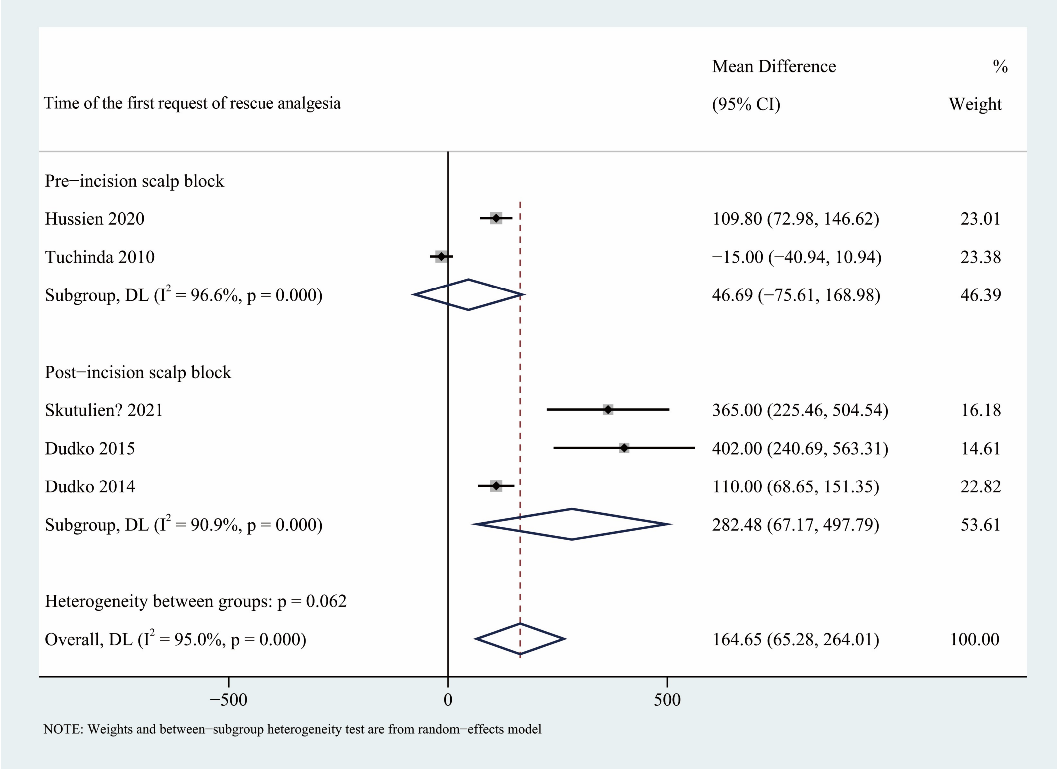

Supplemental Figure 6. Forest plot summarizing meta-analysis of studies reporting additional analgesia requirement in first 24h.

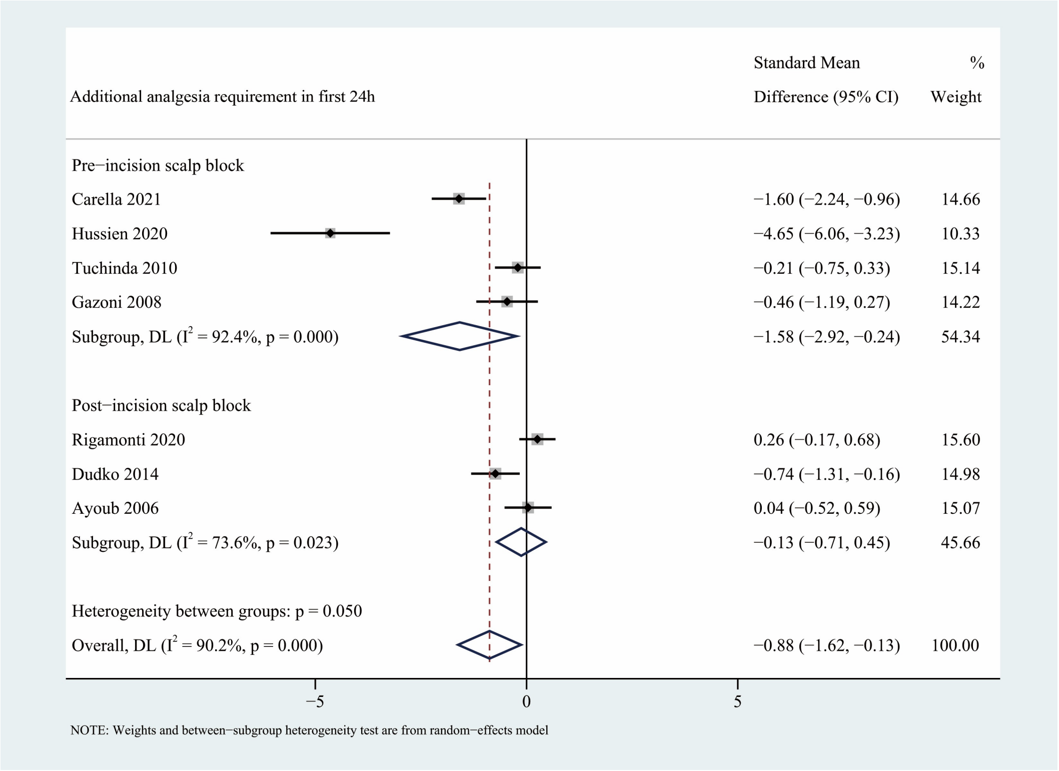

Supplemental Figure 7. Summary of the Egger's publication bias plot.

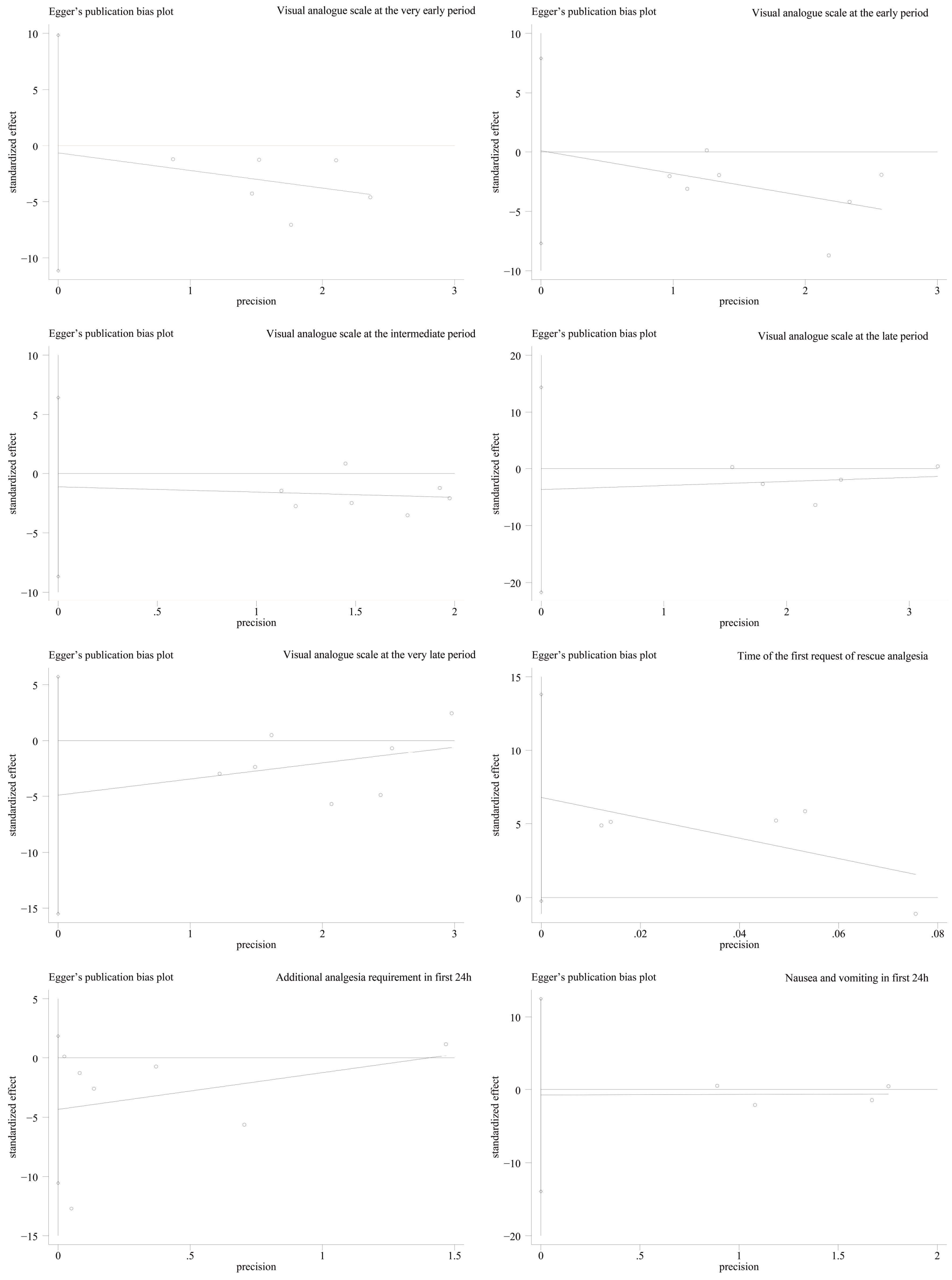

Supplemental Figure 8. "Leave-one-out" sensitivity analysis of studies reporting very early pain score.

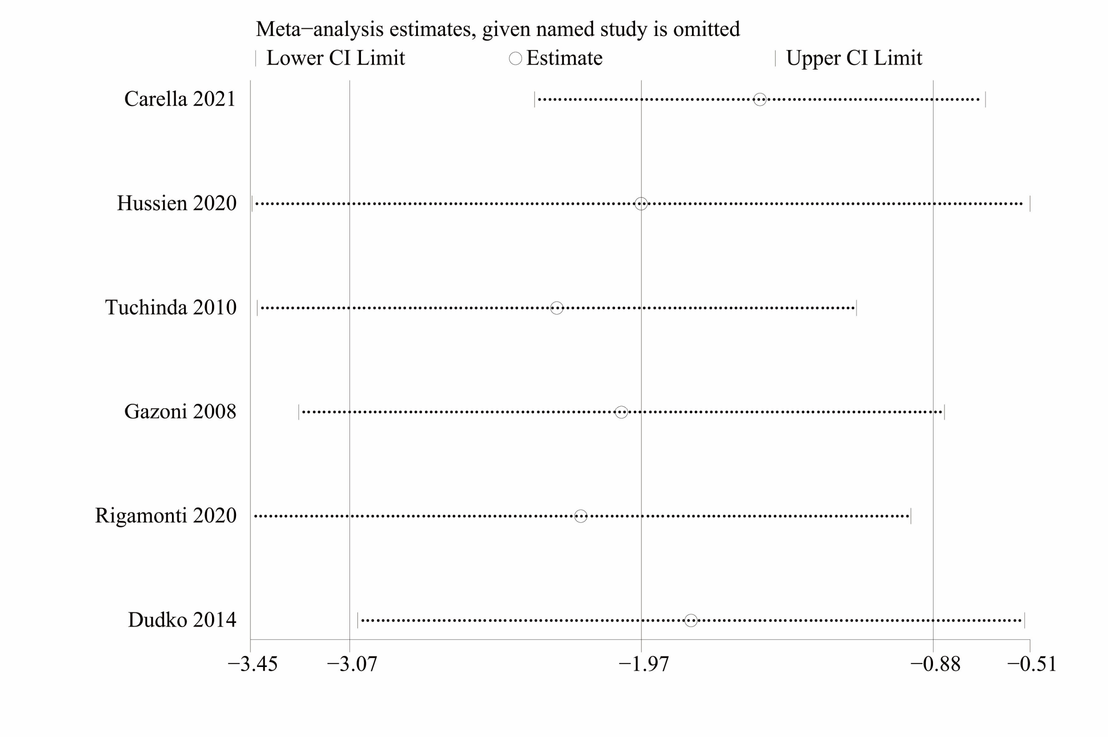

Supplemental Figure 9. "Leave-one-out" sensitivity analysis of studies reporting early pain score.

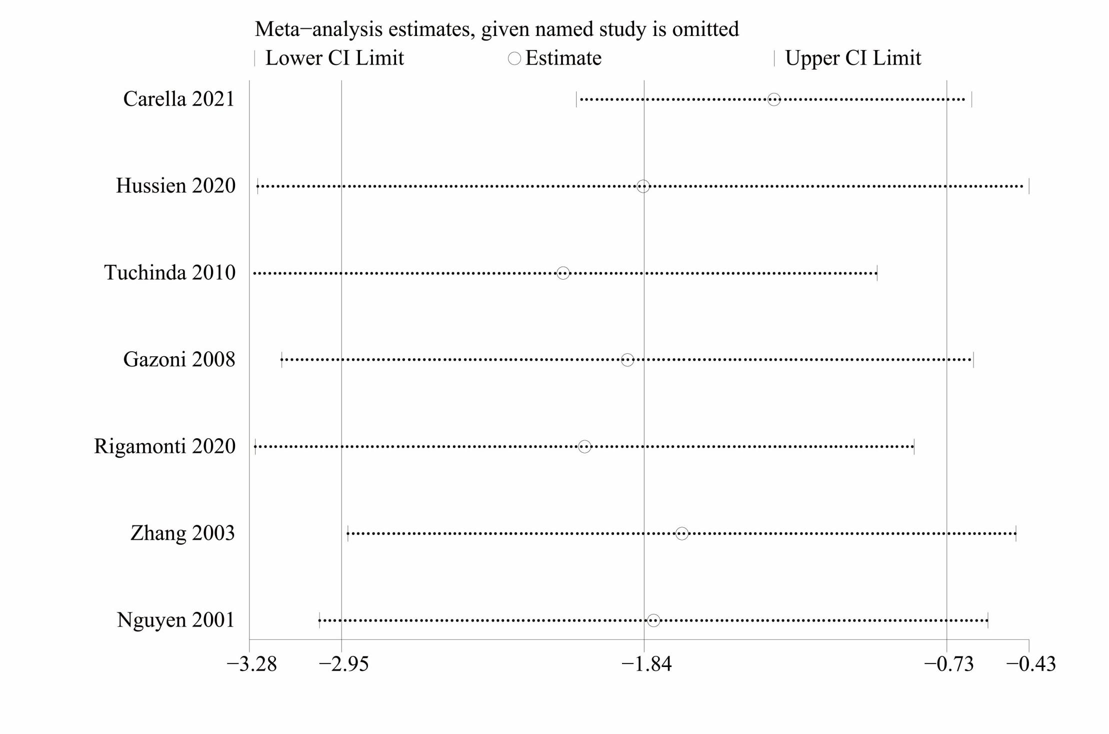

Supplemental Figure 10. "Leave-one-out" sensitivity analysis of studies reporting intermediate pain score.

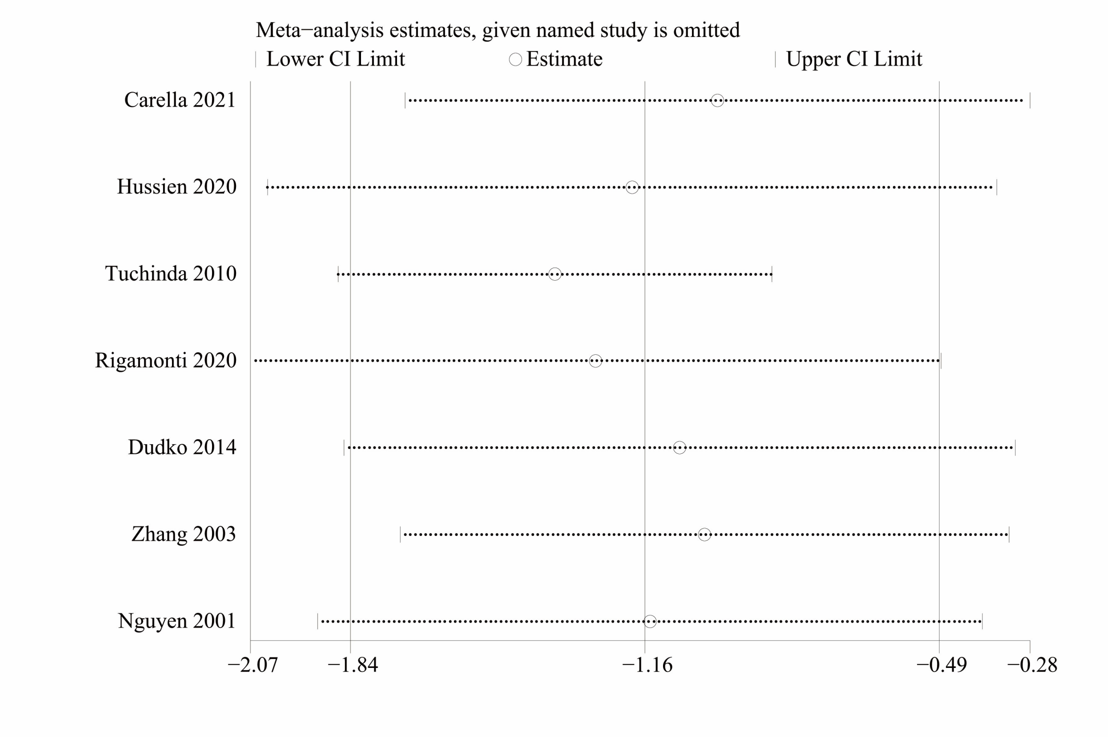

Supplemental Figure 11. "Leave-one-out" sensitivity analysis of studies reporting late pain score.

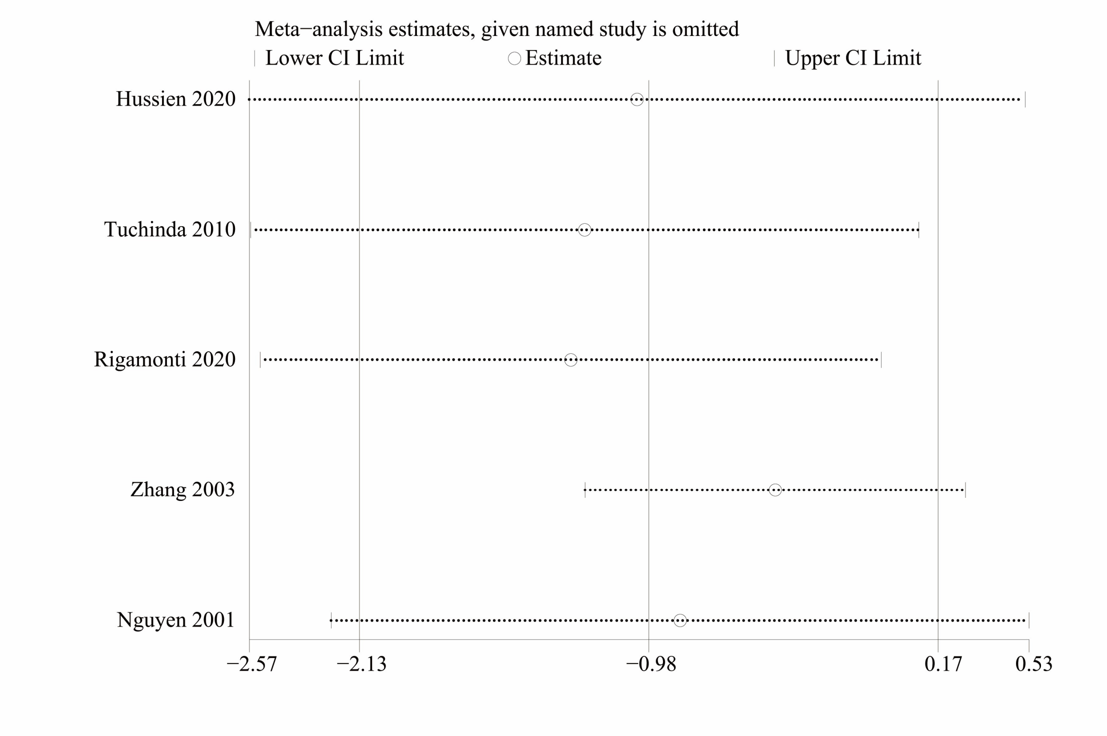

Supplemental Figure 12. "Leave-one-out" sensitivity analysis of studies reporting very late pain score.

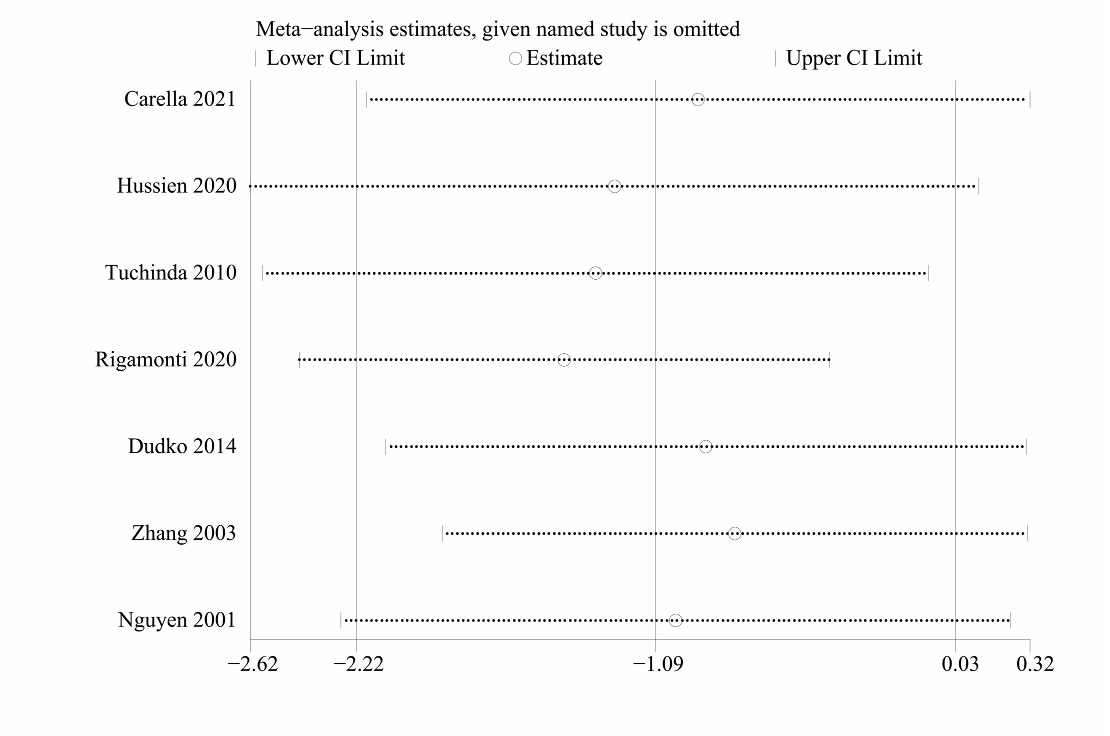

Supplemental Figure 13. "Leave-one-out" sensitivity analysis of studies reporting time of the first request of rescue analgesia.

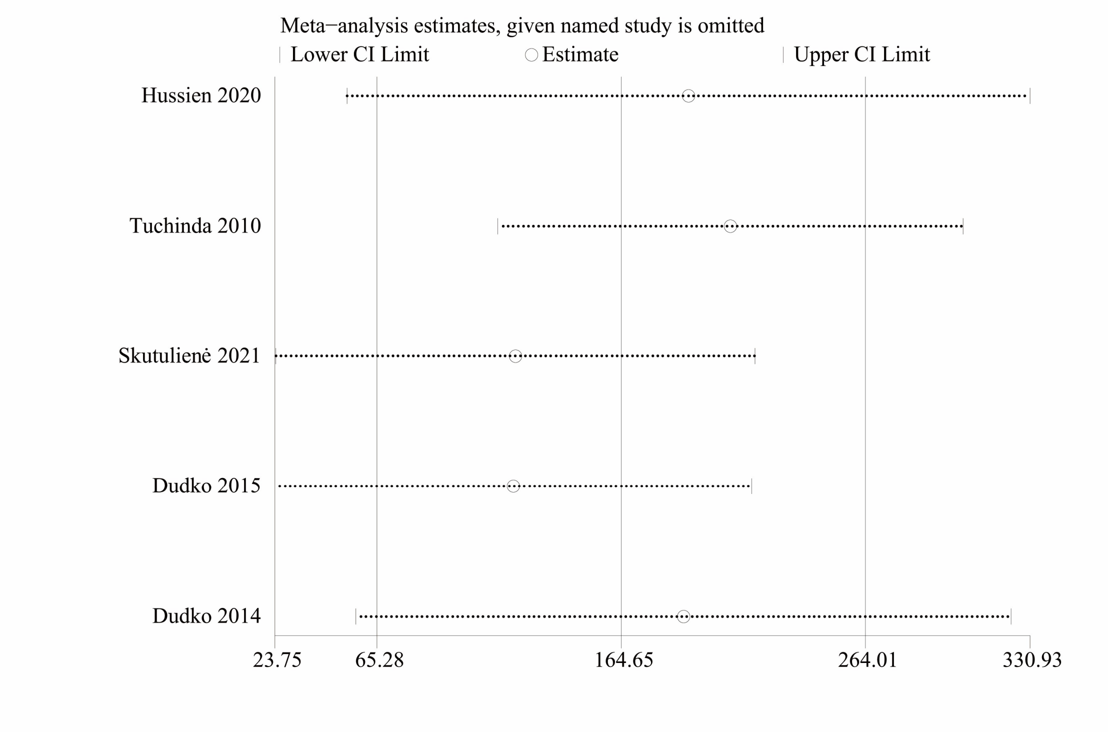

Supplemental Figure 14. "Leave-one-out" sensitivity analysis of studies reporting additional analgesia requirement in first 24h.

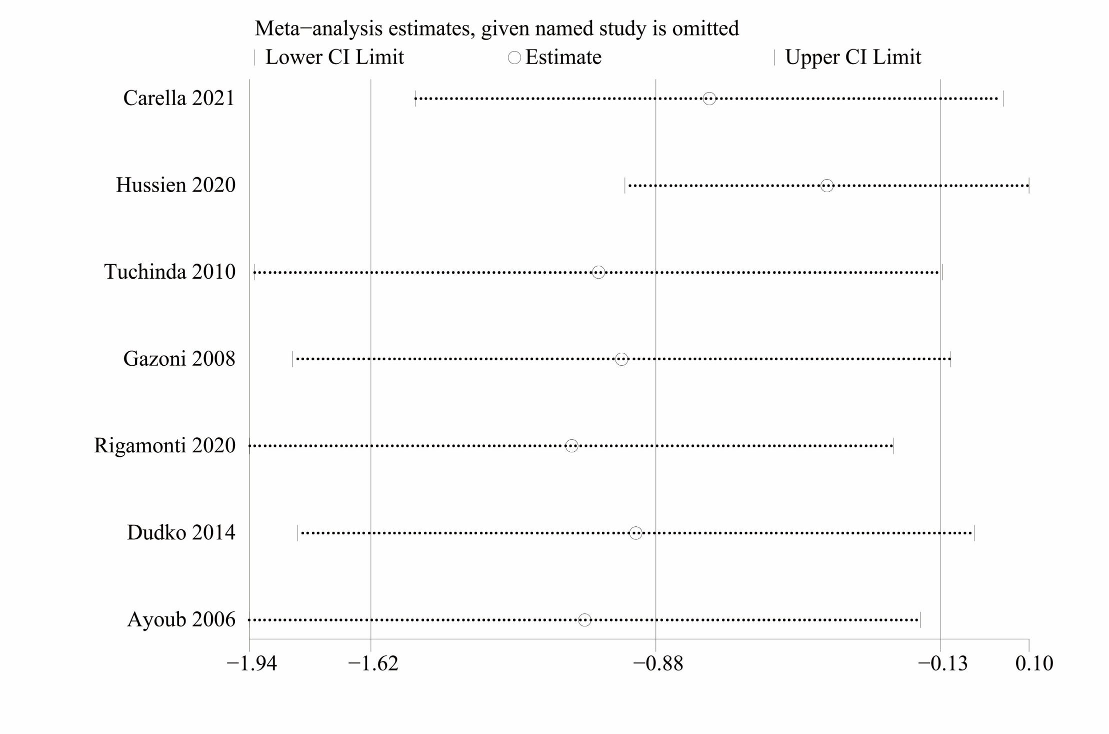

Supplemental Figure 15. "Leave-one-out" sensitivity analysis of studies reporting nausea and vomiting in first 24h.

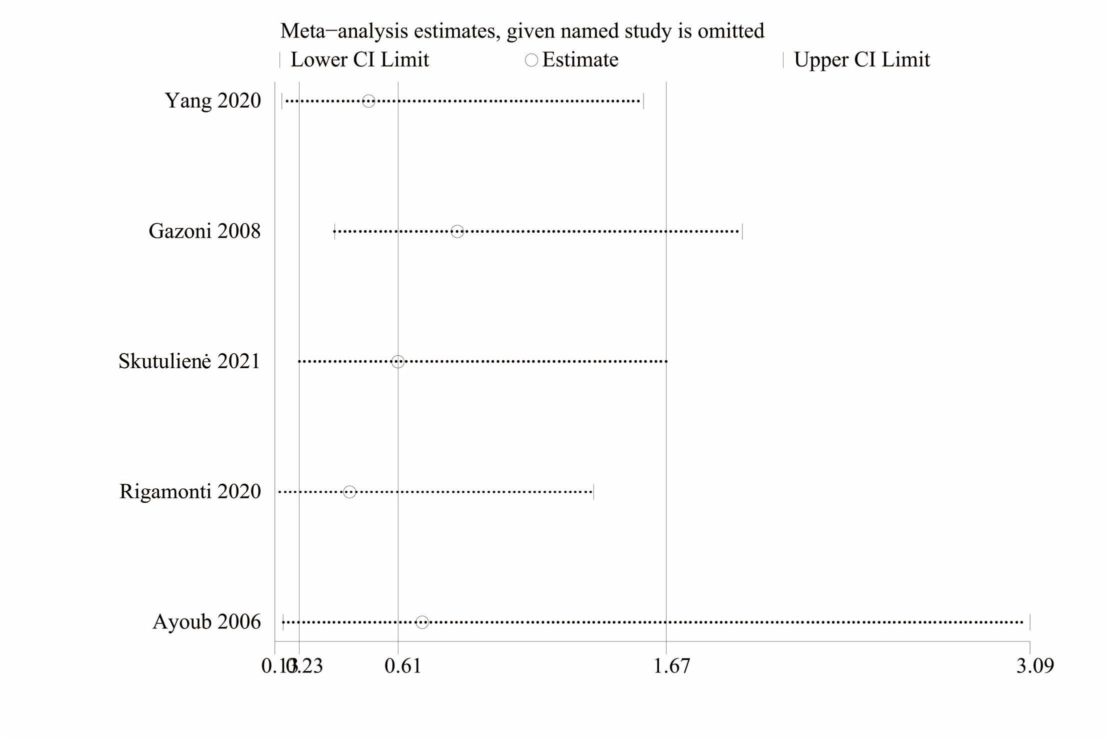

Characteristics of included studies (order by year of publication)

Skutulienė 2021

Methods	Study design: randomized controlled trial (3 arms)<br>Study duration: not reported<br>Study setting: hospital, single center, Lithuanian
Participants	Adults undergoing scheduled supratentorial brain tumor removal (n=141)<br>**Inclusion criteria**<br>1. ASA I–III<br>**Exclusion criteria**<br>1. Glasgow Coma Score (GCS) of less than 15<br>2. Allergy to local anesthetics<br>3. Undergoing long-term analgesic or corticosteroid therapy,<br>4. Cardiac arrhythmias<br>5. Impaired liver function<br>**Mean age (years)**<br>1. 57.2<br>**Numbers allocated to each arm**<br>1. Group wound infiltration (n = 47)<br>2. Group scalp nerve blockade (n = 47)<br>3. Group systemic analgesia (n = 47)<br>**Male gender**<br>1. Group wound infiltration: 13/34<br>2. Group scalp nerve blockade: 15/32<br>3. Group systemic analgesia: 16/31
Interventions	**Technique and occasion**<br>Scalp block of the following nerves with 0.25% bupivacaine combined with 1% lidocaine and 1:200,000 epinephrine after suturing the wound:<br>1. The supraorbital and supratrochlear nerves<br>2. The zygomaticotemporal nerve<br>3. The auriculotemporal nerve<br>4. The greater and lesser occipital nerves<br>**Dosage**<br>20 mL
Outcomes	**Primary**<br>1. Pain as measured by the visual analogue score during the first 24 hours postoperatively (measured at 1, 3, 6 and 24 hours)<br>**Secondary**<br>1. The duration for the request of additional analgesics<br>2. Adverse effects<br>3. Baseline hemodynamic variables during induction through operation and postoperatively
Notes	**Funding**<br>No funding source reported

Carella 2021

Methods	Study design: randomized controlled trial (2 arms)<br>Study duration: October 2016 to December 2019<br>Study setting: hospital, single center, Belgium
Participants	Adults undergoing scheduled supratentorial brain tumor removal (n=60)<br>**Inclusion criteria**<br>1. ASA I–III<br>**Exclusion criteria**<br>1. Allergy to local anesthetic<br>2. Psychiatric disease<br>3. Inability to consent<br>4. Uncontrolled intracranial hypertension<br>**Mean age (years)**<br>1. 57<br>**Numbers allocated to each arm**<br>1. Group SB (n = 30)<br>2. Group CO (n = 30)<br>**Male gender**<br>1. Group SB: 13/17<br>2. Group CO: 13/17
Interventions	**Technique and occasion**<br>Scalp block of the following nerves with 0.33% levobupivacaine after induction of general anesthesia:<br>1. The supraorbital and supratrochlear nerve<br>2. The auriculotemporal nerve<br>3. The postauricular branches of the greater auricular nerve<br>4. The zygomaticotemporal nerve<br>5. The greater, lesser, and third occipital nerves<br>**Dosage**<br>30 mL
Outcomes	**Primary**<br>1. Hemodynamic stability<br>**Secondary**<br>1. Cumulative intraoperative remifentanil consumption<br>2. Cumulative postoperative morphine consumption<br>3. Postoperative pain scores
Notes	**Funding**<br>No funding source reported

Yang 2020

Methods	Study design: randomized controlled trial (4 arms)<br>Study duration: October 2016 to December 2019<br>Study setting: hospital, single center, Belgium
Participants	Adults aged 18 to 60 years, who were waiting for elective craniotomy that acquired general anesthesia (n=85)<br>**Inclusion criteria**<br>1. ASA I–II<br>2. Body mass index 18 to 30 kg/m²<br>**Exclusion criteria**<br>1. Unable to understand or use VAS<br>2. Allergic to local anesthetics<br>3. Glasgow coma scale scores <15<br>4. History of opioid dependence, coagulopathy, scalp infection, pregnancy and previous craniotomy<br>**Mean age (years, Mean±SD)**<br>1. 44±12<br>**Numbers allocated to each arm**<br>1. Group $R_{0.2}$ (n = 21)<br>2. Group $R_{0.33}$ (n = 20)<br>3. Group $R_{0.5}$ (n = 22)<br>4. Group C (n = 22)<br>**Male gender**<br>1. Group $R_{0.2}$: 11/10<br>2. Group $R_{0.33}$: 12/8<br>3. Group $R_{0.5}$: 11/11<br>4. Group C: 7/15
Interventions	**Technique and occasion**<br>Scalp block of the following nerves with 0.33%, 0.33% or 0.5% levobupivacaine before surgical incision and after intubation:<br>1. Unilateral supraorbital, auriculotemporal and lesser occipital nerve of the side of craniotomy<br>2. Bilateral greater occipital nerve<br>**Dosage**<br>8 mL
Outcomes	**Primary**<br>1. Pain as measured by the visual analogue score during the first 24 hours postoperatively (measured at 2, 4, 6 and 24 hours)<br>**Secondary**<br>1. Intraoperative hemodynamic variables (MAP and HR)<br>2. Additional sufentanyl requirements<br>3. Total consumption of dezocine during the first 24 hours after surgery<br>4. The time to first injection<br>5. Incidence of postoperative nausea and vomiting (PONV)<br>6. Complications both from local anesthetic and the nerve block
Notes	**Funding**<br>No funding source reported

Rigamonti 2020

Methods	Study design: randomized controlled trial (2 arms)<br>Study duration: March 2010 to December 2011<br>Study setting: hospital, single center, Canada
Participants	Adults aged 18 years and over, scheduled for supratentorial craniotomy (n=89)<br>**Inclusion criteria**<br>1. ASA physical status < IV<br>**Exclusion criteria**<br>1. History of significant coronary artery disease<br>2. Presence of pre-existing pain related to the Intracranial pathology<br>3. Active history of alcohol or recreational drug abuse<br>4. Active history of psychotic disorder<br>**Mean age (years, Mean±SD)**<br>1. 54.5±15<br>**Numbers allocated to each arm**<br>1. Group treatment (n = 44)<br>2. Group control (n = 45)<br>**Male gender**<br>1. Group treatment: 16/28<br>2. Group control: 24/21
Interventions	**Technique and occasion**<br>Scalp block of the following nerves with 0.5% bupivacaine and 1:200,000 epinephrine at the end of the procedure:<br>1. The supraorbital and supratrochlear nerves<br>2. The auriculotemporal nerve<br>3. The postauricular branches of the greater auricular nerve<br>4. The greater, lesser, and third occipital nerves<br>**Dosage**<br>20 mL
Outcomes	**Primary**<br>1. Pain as measured by the visual analogue score during the first 48 hours postoperatively (measured at 0.5, 1, 2, 4, 8, 12, 24 and 48 hours)<br>**Secondary**<br>1. The total PCA hydromorphone consumption in the first at 24 and 48 post-operative hours<br>2. Total hydromorphone demands and delivered doses in the first 24 and 48 post-operative hours<br>3. The incidence of nausea and vomiting in the first 24 and 48 post-operative hours<br>4. The time for patients to reach discharge eligibility from the PACU/ICU<br>5. The time for patients to reach discharge eligibility from hospital<br>6. Presence of long term pain as measured with the Numeric Rating Scale (NRS) at days 5, 30 and 60 postoperatively<br>7. Karnofsky Performance Scale Index and modified pain treatment satisfaction scale (PTSS) at day 5
Notes	**Funding**<br>This study was supported by the Physicians Services Incorporated Grant (PSI 09-22, PI Andrea Rigamonti).

Hussien 2020

Methods	Study design: randomized controlled trial (2 arms)<br>Study duration: March 2018 to December 2020<br>Study setting: hospital, single center, Egypt
Participants	Patients aged 21–60 years of both genders and prepared to undergo craniotomy under general anesthesia for supratentorial tumors. (n=30)<br>**Inclusion criteria**<br>1. ASA grade I-II<br>2. Body mass index <35 kg/m^2<br>**Exclusion criteria**<br>1. Glasgow coma score < 14<br>2. Huge tumor with marked midline shift And incision extending beyond the areas covered by regional scalp block<br>3. Uncontrolled hypertension<br>**Mean age (years, Mean±SD)**<br>1. 54.5±15<br>**Numbers allocated to each arm**<br>1. Group treatment (n = 15)<br>2. Group control (n = 15)<br>**Male gender**<br>1. Group treatment: 10/5<br>2. Group control: 11/4
Interventions	**Technique and occasion**<br>Scalp block of the following nerves with 0.5% bupivacaine, 2% lidocaine and 1:200,000 epinephrine before skull pinning:<br>1. The supraorbital and supratrochlear nerves<br>2. The auriculotemporal nerve<br>3. The postauricular branches of the greater auricular nerve<br>4. The greater, lesser, and third occipital nerves<br>5. The zygomaticotemporal nerve<br>**Dosage**<br>17 mL
Outcomes	**Primary**<br>1. Intra-operative Heart rate (HR) at different times<br>2. Intraoperative mean arterial pressure changes<br>**Secondary**<br>1. Pain as measured by the visual analogue score during the first 24 hours postoperatively (measured at 0.5, 1, 2, 4, 8, 16 and 24 hours)<br>2. Time from extubation to the first request of analgesia<br>3. Total dose of postoperative Fentanyl consumption in the first 24 hours
Notes	**Funding**<br>No funding source reported

Dudko 2015

Methods	Study design: randomized controlled trial (3 arms)<br>Study duration: not reported<br>Study setting: hospital, single center, Lithuania
Participants	Adults aged 18 years and over, scheduled for supratentorial craniotomy under general anesthesia (n=120)<br>**Inclusion criteria**<br>1. ASA status I-III<br>**Exclusion criteria**<br>Not reported<br>**Mean age (years, Mean±SD)**<br>Not reported<br>**Numbers allocated to each arm**<br>1. Group B (n =40)<br>2. Group I (n = 40)<br>3. Group S (n = 40)<br>**Male gender**<br>Not reported
Interventions	**Technique and occasion**<br>Scalp block with 0.25% bupivacaine, 1% lidocaine and 1:200.000 adrenaline after skin closure<br>**Dosage**<br>Not reported
Outcomes	**Primary**<br>1. Pain as measured by the visual analogue score during the first 24 hours postoperatively<br>**Secondary**<br>1. Administered ketorolac doses<br>2. Duration for the requirement of first rescue analgesia
Notes	**Funding**<br>No funding source reported

Dudko 2014

Methods	Study design: randomized controlled trial (3 arms)<br>Study duration: not reported<br>Study setting: hospital, single center, Lithuania
Participants	Adults aged 18 years and over, scheduled for supratentorial craniotomy under general anesthesia (n=75)<br>**Inclusion criteria**<br>1. ASA status I-III<br>**Exclusion criteria**<br>Not reported<br>**Mean age (years, Mean±SD)**<br>Not reported<br>**Numbers allocated to each arm**<br>1. Group B (n =25)<br>2. Group I (n = 25)<br>3. Group S (n = 25)<br>**Male gender**<br>Not reported
Interventions	**Technique and occasion**<br>Scalp block with 0.25% bupivacaine, 1% lidocaine and 1:200.000 adrenaline after skin closure<br>**Dosage**<br>20ml
Outcomes	**Primary**<br>1. Pain as measured by the visual analogue score during the first 24 hours postoperatively (measured at 1, 3, 6 and 24 hours)<br>**Secondary**<br>1. Administered ketorolac doses<br>2. Duration for the requirement of first rescue analgesia
Notes	**Funding**<br>No funding source reported

Tuchinda 2010

Methods	Study design: randomized controlled trial (3 arms)<br>Study duration: not reported<br>Study setting: hospital, single center, Thailand
Participants	Patients aged 16 to 65 years undergoing elective supratentorial craniotomy (n=60)<br>**Inclusion criteria**<br>1. ASA I and II<br>**Exclusion criteria**<br>1. Unable to assess pain<br>2. Documented allergy to local anesthetics<br>3. With hypertension<br>4. History of opioid dependence, coagulopathy, scalp infection, and previous craniotomy<br>**Mean age (years, Mean±SD)**<br>1. 34.3±11<br>**Numbers allocated to each arm**<br>1. Group 0.5% bupivacaine (n = 20)<br>2. Group 0.25% bupivacaine (n = 19)<br>3. Group normal saline (n = 20)<br>**Male gender**<br>1. Group 0.5% bupivacaine: 10/11<br>2. Group 0.25% bupivacaine: 8/11<br>3. Group normal saline: 14/6
Interventions	**Technique and occasion**<br>Scalp block of the following nerves with 0.5% or 0.25% bupivacaine and 1:200,000 adrenaline before skull pinning:<br>1. The supraorbital and supratrochlear nerves<br>2. The auriculotemporal nerve<br>3. The greater auricular nerve<br>4. The greater, lesser, and third occipital nerves<br>5. The zygomaticotemporal nerve<br>**Dosage**<br>Not reported
Outcomes	**Primary**<br>1. Pain as measured by the visual analogue score during the first 24 hours postoperatively (measured at 0.5, 1, 1.5, 2, 6, 12 and 24 hours)<br>**Secondary**<br>1. Sedation and nausea vomiting scores and antiemetics given to the patients<br>2. Time from extubation to the first analgesic given<br>3. Total morphine consumption in 24 hours post-operatively
Notes	**Funding**<br>This study was supported by the Ratchadapiseksompotch Fund, Faculty of Medicine, Chulalongkorn University.

Gazoni 2008

Methods	Study design: randomized controlled trial (2 arms)<br>Study duration: not reported<br>Study setting: hospital, single center, America
Participants	Adult patients (aged > 18 years) with a supratentorial brain tumor scheduled for resection. (n=30)<br>**Inclusion criteria**<br>Not reported<br>**Exclusion criteria**<br>1. Pregnancy<br>2. The presence of a preexisting intracranial defect<br>3. Allergy to remifentanil or ropivacaine<br>4. History of malignant hyperthermia.<br>**Mean age (years, Mean±SD)**<br>Not reported<br>**Numbers allocated to each arm**<br>1. Group treatment (n = 14)<br>2. Group control (n = 16)<br>**Male gender**<br>Not reported
Interventions	**Technique and occasion**<br>Scalp block of the following nerves with 0.5% ropivacaine after the induction of anesthesia and endotracheal intubation:<br>1. The supraorbital and supratrochlear nerves<br>2. The auriculotemporal nerve<br>3. The greater, lesser, and third occipital nerves<br>4. The zygomaticotemporal nerve<br>**Dosage**<br>Not reported
Outcomes	**Primary**<br>1. BP and HR during the surgery and in the immediate postoperative period<br>2. Intraoperative mean arterial pressure changes total dose of remifentanil and expired concentration of sevoflurane<br>**Secondary**<br>1. Pain as measured by the visual analogue score during the first 4 hours postoperatively (measured at 1, 2 and 4 hours)<br>2. Total opioid consumption<br>3. Incidence of postoperative nausea and vomiting
Notes	**Funding**<br>No funding source reported

Ayoub 2006

Methods	Study design: randomized controlled trial (2 arms)<br>Study duration: not reported<br>Study setting: hospital, single center, Canada
Participants	Patients aged 18–70 years of both genders and scheduled for an elective supratentorial craniotomy. (n=50)<br>**Inclusion criteria**<br>1. ASA grade I-III<br>**Exclusion criteria**<br>1. Inability to understand a numerical rating scale (NRS)<br>2. Proven or suspected allergy to local anesthetics or morphine<br>3. A craniotomy incision extending beyond the field covered by the SNB<br>4. Chronically treated with opioid medications (>2 week)<br>5. Presenting with a history of alcohol abuse and with active psychiatric disorders<br>**Mean age (years, Mean±SD)**<br>1. 50.5±13.4<br>**Numbers allocated to each arm**<br>1. Group block (n = 25)<br>2. Group morphine (n = 25)<br>**Male gender**<br>1. Group block: 14/11<br>2. Group morphine: 15/10
Interventions	**Technique and occasion**<br>Scalp block of the following nerves with 0.5% bupivacaine and 2% lidocaine at the end of surgery:<br>1. The supraorbital and supratrochlear nerves<br>2. The auriculotemporal nerve<br>3. The postauricular branches of the greater auricular nerve<br>4. The greater, lesser, and third occipital nerves<br>**Dosage**<br>20 mL
Outcomes	**Primary**<br>1. Pain as measured by the numerical rating scale during the first 24 hours postoperatively (measured at 1, 2,4 ,8, 12, 16 and 24 hours)<br>**Secondary**<br>1. Cumulative doses of codeine<br>2. Incidence of nausea, and vomiting as well as periods of confusion<br>3. Total dose of postoperative Fentanyl consumption in the first 24 hours
Notes	**Funding**<br>This study was supported in part by a Grant from the Canadian Anesthesiologist Society

Zhang 2003

Methods	Study design: randomized controlled trial (4 arms)<br>Study duration: not reported<br>Study setting: hospital, single center, China
Participants	Patients aged 21–60 years of both genders and prepared to undergoing elective supratentorial craniotomy. (n=60)<br>**Inclusion criteria**<br>1. ASA grade I-III<br>**Exclusion criteria**<br>1. Preoperative use of analgesic<br>**Mean age (years, Mean±SD)**<br>Not reported<br>**Numbers allocated to each arm**<br>1. Group control (n = 10)<br>2. Group SNB (n = 17)<br>1. Group WIA (n = 17)<br>2. Group SCPB (n = 16)<br>**Male gender**<br>Not reported
Interventions	**Technique and occasion**<br>Scalp block of the following nerves with 0.75% ropivacaine at skin closure before the patient was awakened:<br>1. The supraorbital and supratrochlear nerves<br>2. The auriculotemporal nerve<br>3. The postauricular branches of the greater auricular nerve<br>4. The greater and lesser nerves<br>**Dosage**<br>Not reported
Outcomes	**Primary**<br>1. Pain as measured by the visual analogue score during the first 48 hours postoperatively (measured at 4, 8, 12, 16, 24 and 48 hours)<br>**Secondary**<br>Not reported
Notes	**Funding**<br>No funding source reported

Nguyen 2001

Methods	Study design: randomized controlled trial (2 arms)<br>Study duration: not reported<br>Study setting: hospital, single center, Canada
Participants	Patients aged 18 to 70 years of both genders and scheduled to undergo a craniotomy for either a supratentorial mass or an aneurysm clipping. (n=30)<br>**Inclusion criteria**<br>1. ASA physical status I–III<br>**Exclusion criteria**<br>1. Inability to understand or incapacity to use the visual analog scale (VAS)<br>2. Proven or suspected allergy to local anesthetics or codeine phosphate<br>3. A craniotomy incision extending beyond the field of the block<br>4. Chronically (more than 2 weeks) treated with narcotic medications<br>**Mean age (years, Mean±SD)**<br>1. 48±10.4<br>**Numbers allocated to each arm**<br>1. Group ropivacaine (n = 15)<br>2. Group saline (n = 15)<br>**Male gender**<br>1. Group ropivacaine: 8/7<br>2. Group saline: 5/10
Interventions	**Technique and occasion**<br>Scalp block of the following nerves with 0.75% ropivacaine at skin closure before the patient was awakened:<br>1. The supraorbital and supratrochlear nerves<br>2. The auriculotemporal nerve<br>3. The postauricular branches of the greater auricular nerve<br>4. The greater, lesser, and third occipital nerves<br>**Dosage**<br>20 mL
Outcomes	**Primary**<br>1. Pain as measured by the visual analogue score during the first 48 hours postoperatively (measured at 4, 8, 12, 16, 20, 24 and 48 hours)<br>**Secondary**<br>1. Glasgow coma score<br>2. Localization of the site of pain<br>3. Cumulative doses of codeine
Notes	**Funding**<br>No funding source reported
